# Editorial: Insights in cardiovascular imaging: 2022

**DOI:** 10.3389/fcvm.2023.1231842

**Published:** 2023-06-26

**Authors:** Sebastian Kelle, Christos V. Bourantas, Grigorios Korosoglou

**Affiliations:** ^1^ Department of Cardiology, Angiology and Intensive Care Medicine, Deutsches Herzzentrum der Charité, Berlin, Germany; ^2^ German Center for Cardiovascular Research (DZHK), Partner Side, Berlin, Germany; ^3^ Department of Cardiology, Barts Heart Centre, Barts Health NHS Trust, London, United Kingdom; ^4^ Centre for Cardiovascular Medicine and Devices, William Harvey Research Institute, Queen Mary University, London, United Kingdom; ^5^ Department of Cardiology, Vascular Medicine and Pneumology, GRN Hospital Weinheim, Weinheim, Germany; ^6^ Weinheim Cardiovascular Imaging Center, Hector Foundation, Weinheim, Germany

**Keywords:** echocardiography, cardiac magnetic resonance (CMR), cardiac computed tomography (CCT), multimodal imaging, tissue characterization, cardiac masses, diagnostic classification, positron emission tomography (PET)

**Editorial on the Research Topic** Insights in cardiovascular imaging: 2022

Technical developments with cardiovascular imaging techniques, such as echocardiography, cardiac magnetic resonance (CMR), cardiac computed tomography angiography (CCTA), and intravascular imaging, including optical coherence tomography (OCT) and intravascular ultrasound (IVUS), have drastically improved both diagnostic classification and risk stratification of patients with cardiac diseases. In addition, cardiovascular imaging enabled a better understanding of cardiac physiology and pathophysiology, providing important links between genotypes and clinical features in patients with cardiomyopathies and other myocardial disorders. Due to the wider availability of both basic and advanced cardiovascular imaging tools, continuous training of cardiologists to use these tools and interpret cardiovascular imaging findings has become essential. Thus, the presentation of cases where cardiovascular imaging helped define the diagnosis and influence the therapeutic management of patients with cardiac diseases is essential, to increase awareness among clinicians about the role of cardiovascular imaging in modern cardiovascular medicine. Hereby, each imaging technique separately or the combination of imaging tools within a multimodality imaging approach may not only provide the correct diagnosis but also aid the monitoring of treatment strategies. This in addition to the continuous spread of knowledge among experts will contribute to more precise diagnostic algorithms, aiding prompter and more efficient subsequent patient management.

In total 28 research papers were published in this Research Topicand herein, the most important and impactful research findings are summarized. Several of the studies used cardiac magnetic resonance (CMR) for the characterization of cardiac disorders. The feasibility of comprehensive CMR using a low-field MR scanner (0.55 T system with an 80 cm bore) was demonstrated by Varghese et al.. The system provided images with a reasonable quality that enable evaluation of cardiac function, perfusion, viability, and tissue mapping. Although future technical developments are still necessary to improve image quality, such clinical CMR protocols may be utilized in patient cohorts with claustrophobia or severe obesity, who cannot undergo CMR examinations in conventional normal or high-field scanners with narrower bores.

The work of Cheng et al., focuses on CMR techniques and especially those that may help to explore myocardial tissue morphology and function on a deeper level. Such CMR sequences, including mapping techniques and myocardial strain, can detect impaired microvascular integrity and early-stage myocardial fibrosis even without the need for contrast agent injection and within faster CMR scan protocols. Which types of heart disease will profit from such deeper phenotyping and if the latter can impact outcomes in patient care, merits further investigation in future studies.

Four studies of our special issue focused on the myocardial strain assessed by CMR. Hashemi et al., demonstrated for the first time that patients without heart failure symptoms but at increased cardiovascular (CV) risk, demonstrate features seen in patients with heart failure with preserved ejection fraction (HFpEF), such as impaired myocardial deformation indexes at rest but normal strain during handgrip stress. Such individuals may lack clinical symptoms due to the compensation capacities of the myocardium and increased myocardial deformation during stress. This novel finding may imply that some patients at increased CV risk (stage A of heart failure by current guidelines) are candidates for developing HFpEF, which triggers the question if such individuals would be good candidates for preventive heart failure treatments in future pharmacological trials. Strain acquisitions were performed using strain-encoded MR (SENC) in this trial, which has been used in several previous studies for the diagnostic classification and risk stratification of patients with ischemic heart disease and non-ischemic cardiomyopathies (1–4). Basically, phase-based SENC and cine-based feature tracking imaging (FTI) techniques are currently available for quantification analysis of myocardial deformation. Both techniques have advantages and drawbacks. FTI obviates the need for additional acquisitions because strain assessment can be performed using standard cine images. FTI-derived strain is widely available and its ability for the diagnosis of ischemia and for the risk stratification of patients with cardiomyopathies has been demonstrated in previous studies (5–7). SENC on the other hand, provides a single heart-beat evaluation of myocardial strain with high reproducibility, especially in terms of regional analysis (1, 4).

Using a similar methodology, Hashemi et al., also demonstrated that myocardial deformation measurements by SENC are associated with the functional capacity of patients with HFpEF and of those with heart failure with reduced ejection fraction (HFrEF) as well as with quality-of-life measures. In addition, the amount of preserved myocardial contractility by SENC enabled discrimination between symptomatic and asymptomatic individuals, even in those with normal LV-ejection fraction.

In another study, Gräni et al., used feature tracking imaging (FTI) to assess segmental myocardial strain in patients who underwent primary percutaneous coronary intervention (PCI) due to ST-elevation myocardial infarction (STEMI). Segments with late gadolinium enhancement (LGE) and additional microvascular obstruction (MVO) exhibited reduced strain; in this setting, FTI enabled the identification of such segments with MVO with relatively good diagnostic accuracy. Thus, a segmental strain may be a useful marker for contrast-free and fast detection of segments with a higher probability to remain dysfunctional during follow-up, aiding the early risk stratification of patients with STEMI. Using a similar methodology, Liu et al., combined myocardial strain by FTI and LGE imaging to differentiate patients with hypertrophic cardiomyopathy (HCM) from those with hypertensive heart disease (HHD). Based on multivariable regression analysis, a combined model of global radial strain by FTI and LGE allowed accurate classification of these two distinct clinical entities. This study may have important implications for patient care since therapeutic management largely varies between HCM and HHD.

Another CMR study, by Massalha et al., investigated the presence of post-infarction pericarditis, diagnosed by pericardial LGE in patients with re-perfused STEMI. Pericardial LGE was present in 77.5% of the patients after STEMI and was not related to the presence of myocardial LGE or MVO. Unexpectedly, however, pericardial LGE was associated with a lower MACE rate during the follow-up period. This is an interesting finding, which possibly indicates that myocardial injury may activate the epicardium as a potential source of regenerative or reparative processes, and requires more in-depth analysis in future studies.

In another study, Treiber et al., measured the influence of volume fluctuations due to different hydration statuses on the resultant native T1 values in 2,047 patients from a clinical all-comers CMR registry. For the first time in the current literature, a weak, albeit relevant influence of volume status was noted on native T1. Patients with volume expansion exhibited significantly higher native T1 values than non-volume-overloaded or dehydrated patients. Despite the effect of volume status on native T1, the latter was a predictor of all-cause mortality in this large, all-comer cohort. This is an important article highlighting the capabilities but also limitations of native T1 measures, which need to be considered by clinicians and scientists who study myocardial disorders such as myocarditis, cardiomyopathies, and cardiac amyloidosis. In this regard, an abnormal T1 value in the presence of normal metrics for myocardial morphology and function may not be enough for establishing the diagnosis of a myocardial disorder due to such variations, as described by Treiber et al. Thus native T1 needs to be judged together with other imaging parameters, clinical variables, and biochemical markers and not as a “stand-alone” value.

Morbus Fabry is a relatively rare X-chromosome-linked hereditary disorder that is caused by a defect in the glycosphingolipid metabolism due to deficiency of the α-galactosidase A enzyme. In an overview article, Umer et al., summarized the role of CMR for the diagnostic classification and risk stratification of patients with Fabry disease. Hereby, the versatility of CMR provided an assessment of myocardial function and strain, LGE, native T1 mapping, and extracellular volume (ECV), which along with biomarker testing can effectively detect most cases with early organ involvement due to Fabry disease. In this context, it is important to note that fibrosis by native T1 mapping and LGE may become apparent with Morbus Fabry prior to left ventricular hypertrophy (8). Thus, CMR in conjunction with biochemical markers may aid the early detection of organ involvement in mild Fabry disease, which may improve clinical outcomes by prompter initiation of enzyme replacement therapy in such patients.

In another study, Snel et al., evaluated the ability of several CMR parameters to detect subclinical alterations in cardiac morphology, function, and tissue characteristics in young adults with CV risk factors, such as hypertension, type 2 diabetes mellitus and being overweight. In this study, including 311 subjects, sedentary life-style related risk factors were shown to be associated with subclinical alteration of myocardial function. The clinical impact of this observation and its association with future adverse cardiac events merits further investigation and in-depth analysis in future studies. Similar to the study by Hashemi et al., it will be intriguing to investigate if early lifestyle modification or pharmacological interventions in such patients can reverse myocardial dysfunction and improve patient outcomes.

In a commentary article, Tesche et al., discuss several cardiac computed tomography angiography (CCTA) markers, such as epicardial adipose tissue, pericoronary adipose tissue fat attenuation, and high-risk coronary plaque features such as positive remodeling, spotty calcifications, low-attenuation plaques, and the napkin-ring sign, including their role as potential precursors of plaque rupture causing acute coronary events. Epicardial adipose tissue (EAT) was recently shown to be associated with low-attenuation plaque burden (Yamaura et al.), which is a known predictor of MACE. Indeed, a growing body of evidence supports the value of EAT as a surrogate marker of coronary wall inflammation, potentially triggering plaque progression and destabilization (9). In addition, EAT has been shown to be associated with non-calcified atherosclerotic plaque burden and increased risk for future cardiac events (10, 11). Importantly, CCTA can visualize and precisely quantify all these markers within a single examination. The body of evidence is growing in this exciting field of CCTA research while there are ongoing trials that are investigating in parallel the effects of lipid-lowing therapies on plaque progression using serial CCTA imaging (DRKS-ID-DRKS00031954). In addition, modern photon counting detectors represent a novel technological achievement, which provides multi-energy capabilities, increased spatial resolution, and tissue contrast with simultaneously reduced radiation exposure for the patients. With this new technology, numerous limitations of traditional CCTA imaging, such as blooming artifacts with strongly calcified plaques and beam-hardening artifacts with coronary stents, can be improved or even completely overcome (12). Plaque characterization and assessment of EAT and peri-coronary tissue may also improve, providing even more accurate risk stratification in patients with CAD.

Another CT study, by Sharkey et al., introduced a nine-structure segmentation model of the heart and great vessels, which was based on a convolutional neural network. This model was tested in patients with pulmonary artery disease and volumetric imaging was correlated with invasive hemodynamic measures. The automated segmentation algorithm enabled overall accurate segmentation of anatomical structures and failed only in cases of poor contrast opacification. Despite the observed limitations, this article demonstrates the potential of automated segmentation, which may be equal to or even outperform human volume measurements in future studies.

Finally, the role of artificial intelligence (AI) in cardiovascular imaging is highlighted by Wellnhofer et al. The authors anticipate that advances in computing power in combination with novel AI algorithms for medical applications will largely impact future patient care. The need for high-quality image data to achieve good machine learning practices and novel regulatory approaches is thoroughly discussed and ethical and legal concerns are presented in this context.

Advances in cardiac imaging are of great clinical importance in current practice. Due to the plethora of imaging techniques and the associated costs, expansion of the diagnostic capabilities of each modality, and selection of the right imaging technique that will enable complete assessment of heart pathology is essential for optimal patient care. All the articles included in this Research Topic demonstrate how cardiovascular imaging can aid the diagnostic classification and risk stratification of patients with a wide variety of cardiac diseases. Standardization and harmonization of image analysis and interpretation among cardiologists and cardiac imagers need to be incorporated into the daily clinical practice, helping to further improve patient care and clinical outcomes.

## References

[1] Steen H Montenbruck M Kelle S Esch S Schwarz AK Giusca S Fast-strain encoded cardiac magnetic resonance during vasodilator perfusion stress testing. Front Cardiovasc Med. (2021) 8:765961. 10.3389/fcvm.2021.765961 34869679 PMC8635645

[2] Korosoglou G Giusca S Montenbruck M Patel AR Lapinskas T Götze C Fast strain-encoded cardiac magnetic resonance for diagnostic classification and risk stratification of heart failure patients. JACC Cardiovasc Imaging. (2021) 14(6):1177–88. 10.1016/j.jcmg.2020.10.024 33454266

[3] Korosoglou G Giusca S Hofmann NP Patel AR Lapinskas T Pieske B Strain-encoded magnetic resonance: a method for the assessment of myocardial deformation. ESC Heart Fail. (2019) 6(4):584–602. 10.1002/ehf2.12442 31021534 PMC6676282

[4] Giusca S Korosoglou G Zieschang V Stoiber L Schnackenburg B Stehning C Reproducibility study on myocardial strain assessment using fast-SENC cardiac magnetic resonance imaging. Sci Rep. (2018) 8(1):14100. 10.1038/s41598-018-32226-3 30237411 PMC6147889

[5] Buss SJ Breuninger K Lehrke S Voss A Galuschky C Lossnitzer D Assessment of myocardial deformation with cardiac magnetic resonance strain imaging improves risk stratification in patients with dilated cardiomyopathy. Eur Heart J Cardiovasc Imaging. (2015) 16(3):307–15. 10.1093/ehjci/jeu181 25246506

[6] Korosoglou G Giusca S. Strain for stress testing: is it a featuring or supporting role in cardiac magnetic resonance? JACC Cardiovasc Imaging. (2020) 13(1 Pt 1):66–8. 10.1016/j.jcmg.2019.04.006 31103585

[7] Romano S Romer B Evans K Trybula M Shenoy C Kwong RY Prognostic implications of blunted feature-tracking global longitudinal strain during vasodilator cardiovascular magnetic resonance stress imaging. JACC Cardiovasc Imaging. (2020) 13(1 Pt 1):58–65. 10.1016/j.jcmg.2019.03.002 31005520 PMC6745296

[8] Weidemann F Beer M Kralewski M Siwy J Kampmann C. Early detection of organ involvement in fabry disease by biomarker assessment in conjunction with LGE cardiac MRI: results from the SOPHIA study. Mol Genet Metab. (2019) 126(2):169–82. 10.1016/j.ymgme.2018.11.005 30594474

[9] Guglielmo M Lin A Dey D Baggiano A Fusini L Muscogiuri G Epicardial fat and coronary artery disease: role of cardiac imaging. Atherosclerosis. (2021) 321:30–8. 10.1016/j.atherosclerosis.2021.02.008 33636676

[10] Gitsioudis G Schmahl C Missiou A Voss A Schüssler A Abdel-Aty H Epicardial adipose tissue is associated with plaque burden and composition and provides incremental value for the prediction of cardiac outcome. A clinical cardiac computed tomography angiography study. PLoS One. (2016) 11(5):e0155120. 10.1371/journal.pone.0155120 27187590 PMC4871366

[11] Brandt V Bekeredjian R Schoepf UJ Varga-Szemes A Emrich T Aquino GJ Prognostic value of epicardial adipose tissue volume in combination with coronary plaque and flow assessment for the prediction of major adverse cardiac events. Eur J Radiol. (2022) 148:110157. 10.1016/j.ejrad.2022.110157 35063819

[12] Cademartiri F Meloni A Pistoia L Degiorgi G Clemente A Gori C Dual-Source photon-counting computed tomography-part I: clinical overview of cardiac CT and coronary CT angiography applications. J Clin Med. (2023) 12(11):3627. 10.3390/jcm12113627 37297822 PMC10253312

